# Acute Kidney Injury in Lymphoma: A Single Centre Experience

**DOI:** 10.1155/2014/272961

**Published:** 2014-02-03

**Authors:** Muhammad Abdul Mabood Khalil, Hira Latif, Abdur Rehman, Waqar Uddin Kashif, Safia Awan, Zarghona Khalil, Uziar Mushtaq, Maria Ahmad, Muhammad Ashhad Ullah Khalil, Manickam Ranga Sami, Jackson Tan

**Affiliations:** ^1^Section of Nephrology, Department of Medicine, Aga Khan University Hospital, Karachi 74800, Pakistan; ^2^Department of Medicine, Khyber Teaching Hospital, Peshawar 25000, Pakistan; ^3^Department of Nephrology, SSB Hospital, Kuala Belait KA1131, Brunei Darussalam; ^4^Department of Nephrology, RIPAS Hospital, Bandar Seri Begawan BA1710, Brunei Darussalam

## Abstract

*Background*. Acute kidney injury (AKI) is a common but least studied complication of lymphoma. *Objective*. To determine the frequency and predictors of AKI in lymphoma and to study the impact of AKI on hospital stay and mortality. *Methods*. Retrospective review of medical records of hospitalized lymphoma patients aged ≥14 years between January 2008 and December 2011 was done. *Results*. Out of 365 patients, AKI was present in 31.8% (116/365). Multivariate logistic regression analysis showed that independent predictors for AKI included sepsis (odds ratio (OR) 3.76; 95% CI 1.83–7.72), aminoglycosides (OR 4.75; 95% CI 1.15–19.52), diuretics (OR 2.96; 95% CI 1.31–6.69), tumor lysis syndrome (OR 3.85; 95% CI 1.54–9.59), and R-CVP regimen (OR 4.70; 95% CI 1.20–18.36). AKI stages 2 and 3 was associated with increased hospital stay (OR 2.01; 95% CI 1.19–3.40). *Conclusion*. AKI was significantly associated with sepsis, aminoglycoside, diuretics, presence of tumor lysis syndrome, and use of R-CVP regimen. Presence of AKIN (Acute Kidney Injury Network) stages 2 and 3 AKI had increased hospital stay. AKI was also associated with increased mortality.

## 1. Introduction

Acute kidney injury (AKI) is a well-known complication of cancer [1, 2]. Etiology of AKI in cancer varies from acute tubular necrosis due to medication or sepsis, volume depletion, tumor lysis syndrome, and obstruction or infiltration of the kidneys. Presence of AKI leads to longer hospitalization and increased cost [3, 4]. It also increases mortality in patients with cancer [1, 2], delays treatment, and increases toxicity of chemotherapy.

Like all cancers, AKI can also occur in lymphoma. The literature reports on AKI in lymphoma are limited to case reports. Lymphoma patients are prone to develop AKI due to acute tubular necrosis secondary to sepsis, nephrotoxic medications, and contrast studies. Lymphomatous infiltration of kidneys [5, 6], light chain proximal tubulopathy [7], membranous nephritis [8], membranoproliferative glomerulonephritis [9], Fanconi syndrome [10], and tumor lysis syndrome [11–13] have all been reported in the literature. This is the first study done on AKI in lymphoma patients and aims to determine frequency and predictors of AKI in lymphoma and to study its impact on hospital stay and mortality.

## 2. Materials and Methods

### 2.1. Setting and Study Population

This is a retrospective case series study conducted at the Aga Khan University Hospital Karachi, Pakistan. Aga Khan University Hospital is a tertiary care health facility located in the largest city of Pakistan, Karachi, which has a population of around 15 million. It has 15 inpatient units with a total capacity of 563 beds. Data were collected upon approval from the ethics review committee of the institution. Study subjects included inpatients admitted with a primary diagnosis of lymphoma. They were identified from a central computerized record for a period of 4 years from January 2008 to December 2011 with the help of the Department of Health Information Management System. Records of the cases were retrieved through codes using International Classification of Diseases 9th Revision Clinical Modification (ICD-9 CM) 201.90–201.98 for Hodgkin lymphoma and 202.80–202.88 for non-Hodgkin lymphoma. Study subjects included patients aged ≥14 years admitted with the primary diagnosis of lymphoma, irrespective of type or stage of disease. Patients with chronic kidney disease were excluded. Patients were divided into two cohorts (those with and those without AKI) in order to determine independent predictors of AKI. Similarly, length of stay and mortality inside hospital were also compared in these two cohorts. Data on demographics, clinical features, laboratory data, length of stay, recovery of renal functions, and mortality were noted. We used the Acute Kidney Injury Network (AKIN) definition for classification of AKI stages [14]. AKI stage 1 was defined as an increase in serum creatinine >26.5 *μ*mole/L (≥0.3 mg/dL) or 1.5- to 2-fold increase from baseline. AKI stage 2  was defined as an increase in serum creatinine of >2-3-fold from baseline.  AKI stage 3was defined as an increase in serum creatinine of >3-fold from baseline or an absolute serum creatinine of >354 *μ*mol/L (≥4 mg/dL).

### 2.2. Definitions

Sepsis was defined as suspected or confirmed infection with the presence of two of the following:

1. temperature > 38°C or <36.5°C;
2. heart rate > 90 per minute;
3. respiratory rate > 30 per minute;
4. leukocyte count > 11000 or <4000.

Tumor lysis syndrome (Cairo-Bishop definition) was defined as 2 of the following:

1. uric acid > 8 mg/dL or 25% increase from the baseline;
2. potassium > 6 mmol/L or 25 % increase from the base line;
3. phosphate > 4.5 mg/dL;
4. calcium < 7 mg/dL or 25% decrease from the baseline 3 days before or 7 days afterward.

### 2.3. Data Analysis

Descriptive statistics were used to summarize baseline values and demographic data. Quantitative data were expressed as mean, median, interquartile range and standard deviation (SD) and number of observations with percentage (%), respectively. Evaluation of association between outcomes (AKI and without AKI group, length of hospital stay) and potential causative factors were used with *χ* ^2^-test or Fisher's exact tests of independence and Student's *t*-test. Odds ratios (OR) and their 95% confidence intervals (CI) were estimated using binary logistic regression, with AKI and length of hospital stay (≤7 versus >7 days) as outcomes. Multivariable models were constructed, including variables that showed an effect on the prediction of AKI and length of hospital stay >7 days in the univariate analysis. All *P* values were based on two-sided tests and significance was set at a *P* value <0.05. The analyses were performed using SPSS version 19.

## 3. Results

### 3.1. General Characteristics of the Study Population

Out of 365 patients, two-thirds (71.8%) were male. Mean age was 50.13 ± 16.3 years. One hundred and sixteen (31.8%) patients had AKI. Among patients with AKI, 78 (67.2%) had AKIN stage 1, 25 (21.6%) had AKIN stage 2, and 13 (11.2%) had AKIN stage 3. Sepsis was found in 64 (17.5%) and tumor lysis in 28 (7.7%) of the patients. Furosemide, vancomycin, amphotericin, and aminoglycoside were used in 40 (11%), 34 (9.31%), 16 (4.4%), and 14 (3.8%) patients, respectively. Overall hospital mortality was 9.3% in AKI patients (Table 1).

### 3.2. Independent Predictors for AKI

We analyzed various variables including antimicrobial and chemotherapeutic agents used. Various chemotherapy regimen used included R-CHOP (rituximab-cyclophosphamide, hydroxy daunorubicin, oncovin, and prednisolone), CHOP (cyclophosphamide, hydroxy daunorubicin, oncovin, prednisolone), R-CVP (rituximab-cyclophosphamide, vincristine, and prednisolone), CVP (cyclophosphamide, vincristine, and prednisolone), ABVD (adriamycin, bleomycin, vinblastine, and dacarbazine) and DHAP (dexamethasone, high dose Ara C, and cisplatin). R-CHOP, CHOP, and ABVD were the main regimens used in 94, 86, and 52 patients, respectively. Univariate analysis revealed increased age (*P*  0.005), diuretics (*P* < 0.001), vancomycin (*P*  0.02), aminoglycoside (*P* < 0.001), R-CVP (*P*  0.004), DHAP (*P*  0.009), sepsis (*P* < 0.001), and tumor lysis (*P* < 0.001) as positive predictors of AKI. However, multivariate logistic regression analysis showed that independent predictors for AKI were sepsis (OR 3.76; 95% CI 1.83–7.72), aminoglycosides (OR 4.75; 95% CI 1.15–19.52), tumor lysis syndrome (OR 3.85; 95% CI 1.54–9.59), and R-CVP regimen (OR 4.70; 95% CI 1.20–18.36) (Tables 2 and 3).

### 3.3. Impact on Hospital Stay, Mortality, and Outcome at Discharge

Patients with AKI stages 2 and 3 had statistically increased hospital stay of greater than 7 days when compared to those without AKI (OR 2.01; 95% CI 1.19–3.40). Mortality was also high in patients with AKI (14.7%) when compared to those without AKI (6.8%) (*P*  0.03) (Table 4). Patients with AKI who died were significantly associated with tumor lysis (*P*  0.001), need of mechanical ventilation (*P* < 0.001), diuretics (*P*  0.001), and sepsis (<0.001) (Table 5).

Mortality in our cohort was 14.7%. Forty-three patients (37.06%) with AKI fully recovered. Around 40 patients (34.48%) had persisting AKI at discharge but did not need dialysis. However 14 (12.06%) patients who needed dialysis were still requiring dialysis at the time of discharge (Table 6).

## 4. Discussion

AKI is a well-known complication of cancer. However, very little is known about AKI in lymphoma. To the best of our knowledge, this is the largest report of AKI in lymphoma patients. Our study revealed the presence of AKI in 31.8% of patients. The incidence of AKI in various cancers has been reported to be between 12 and 49% [15–19]. Our study incidence is higher than that of Christiansen et al., who reported incidence of AKI (defined as 50% elevation of baseline serum creatinine) in 29.39% lymphoma patients [20]. The discrepancy could be explained by the noninclusion of milder AKI patients in their study.

Our multivariate analysis identified sepsis, aminoglycoside usage, diuretics usage, tumor lysis syndrome, and R-CVP usage as potential negative predictors. Cancer patients may be immunocompromised due to multiple factors such as chemotherapy, radiotherapy, impairment of normal leukocyte function, or use of corticosteroids [21, 22]. The dysregulation of their immune system predisposes patients to sepsis which can lead to unregulated cytokine release and haemodynamic disturbances. AKI usually ensues as a result of alterations in renal perfusion resulting from pro-inflammatory insults.

Diuretics usage may have a direct role in causing kidney injury by exacerbating underlying hypovolemic and haemodynamic state. It can also be argued that patients requiring diuretic therapy are likely to have compromised heart or renal function at baseline and, therefore, more likely to develop and sustain kidney injury. On the other hand, it is possible that diuretic therapy was initiated to maintain or increase urine output in patients who may already have AKI. Aminoglycoside has been implicated in the development of kidney injury in haematological malignancies. This could be related to its direct nephrotoxic effects or through synergistic nephrotoxic activities with chemotherapy. The presence of sepsis which may have necessitated the usage of aminoglycoside may also have contributed to the risk of AKI.

We found tumor lysis syndrome in 7.7% of our study population. Various studies have reported spontaneous tumor lysis syndrome in leukemia and lymphoma [11, 23–26]. Hande kjellstrand and his colleagues found tumor lysis in 42% of the patients with lymphoma, which is higher than our result [23]. However, unlike our study Hande Kjellstrand et al. included only high grade lymphoma which has a greater propensity for the development of tumor lysis syndrome.

Various chemotherapy regimens were used in our cohort of patients. From our experience, the use of R-CVP regimen was significantly associated with AKI. R-CVP regimen is used for stages III and VI follicular lymphoma. High tumor burden in stages III and IV follicular lymphoma with potential of tumor lysis in combination with cyclophosphamide nephrotoxicity might be a reason for this. Alternatively, combination of cyclophosphamide with vincristine may be more nephrotoxic, which needs further exploration through future studies. AKI in cancers causes significant morbidity and mortality. Lameire et al. reported 3-month mortality of 30 percent [27]. Another study reported six-month mortality of 73% in critically ill patients with cancer and AKI [28]. Lahoti et al. showed relatively mild elevation of creatinine with worse mortality [29]. We in our study found mortality of 14.7% in patients with lymphoma complicated with AKI as compared to 6.8% in those without AKI. Similarly, presence of AKI stages 2 and 3 in patients with lymphoma had more propensity for a prolonged hospital stay.

We speculate that tumor lysis syndrome, concurrent sepsis with superimposed use of aminoglycosides, and diuretics use make lymphoma patients prone to develop AKI. This in turn results in more mortality and morbidity. Therefore, patients with high grade lymphoma should be cautiously watched during their chemotherapy as they can potentially deteriorate by developing tumor lysis and subsequent AKI. Similarly, sepsis should be vigorously looked for and treated appropriately. While managing gram negative sepsis broad spectrum, antibiotics other than aminoglycosides should be chosen if possible. Patients with sepsis and tumor lysis are often volume depleted and need aggressive fluid resuscitation. Therefore, diuretics use should be judiciously used only in the presence of evident fluid overload. We can thus potentially avoid AKI and hence can decrease mortality and morbidity.

We acknowledge several limitations of our study. This is a retrospective study based on data from a single centre. Due to the retrospective nature of this study, etiology of AKI was difficult to determine. Similarly, due to lack of long-term followup of the patients, long-term impact of AKI on lymphoma patients could not be determined. Multicentre prospective studies with long-term followup are needed to analyze etiology of AKI, its long-term outcome, and impact on mortality and morbidity. Similarly, there is a need to determine least nephrotoxic chemotherapy regimen through prospective analysis. Judging by the results of this study, we propose the following management considerations for high-risk lymphoma patients:

1. careful attention to haemodynamics and volume status;
2. avoidance of specific drugs;
3. avoidance of tumour lysis with prophylactic measures (e.g., prophylactic allopurinol/aggressive rehydration, etc.);
4. early identification and treatment of sepsis;
5. choice of chemotherapy;
6. early initiation of RRT (? debatable);
7. early involvement of nephrology team;
8. early involvement of intensivists.

## 5. Conclusions

AKI is common in lymphoma. Tumor lysis syndrome, sepsis, and use of aminoglycoside and furosemide were associated with development of AKI. Presence of AKI stages 2 and 3 resulted in increased mortality and prolonged hospital stay. We feel that identification of these negative predictors and early intervention can help to prevent or reduce the severity of AKI.

## Figures and Tables

**Table 1:** Demographic characteristics of study population (*n* = 365).

Age, years, mean ± SD	50.3 ± 16.3
Gender, number (% )	
Male	262 (71.8)
Female	103 (28.2)
Lymphoma, number (%)	
HL (Hodgkin lymphoma)	87 (23.8)
NHL (non-Hodgkin lymphoma)	278 (76.2)
Mechanical ventilation, number (%)	23 (6.3)
Vasopressors, number (%)	31 (8.5)
Diuretics, number (%)	40 (11)
Vancomycin, number (%)	34 (9.3)
Amphotericin B, number (%)	16 (4.4)
Aminoglycosides, number (%)	14 (3.8)
Median baseline creatinine	0.8 (0.4–11.1)
Median (range) length of stay	2 (0–45)
Renal replacement therapy (%)	12 (3.3)
Tumor lysis syndrome (%)	28 (7.7)
Sepsis	64 (17.5)
AKI	116 (31.8)
	AKI stage 1 = 78 (67.2%)
	AKI stage 2 = 25 (21.6%)
	Aki stage 3 = 13 (11.2%)
Mortality (%)	34 (9.3%)

**Table 2:** Univariate analysis of factors associated with progression of AKI (*n* = 365).

	AKI, *n* = 116	No AKI, *n* = 249	*P* value
Age, in years	53.7 ± 15.5	48.7 ± 16.5	0.005
Gender			
Male	82 (70.7)	180 (72.3)	0.75
Female	34 (29.3)	69 (27.7)	
Hospital stay, median (range)	3 (1–17)	3 (1–14)	0.62*
Baseline Cr, median (range)	0.8 (0.4–2.6)	0.8 (0.4–11.1)	0.39*
Maximum Cr, median (range)	2 (0.9–11.3)	1 (0.6–25.9)	<0.001*
Stage of lymphoma			
I	4 (9.3)	9 (9.1)	0.17
II	7 (16.3)	32 (32.3)	
III	10 (23.3)	24 (24.2)	
IV	22 (51.2)	34 (34.3)	
Diuretics	24 (20.7)	16 (6.4)	<0.001
Vancomycin	17 (14.7)	17 (6.8)	0.02
Amphotericin B	7 (6)	9 (3.6)	0.28
Aminoglycosides	11 (9.5)	3 (1.2)	<0.001
Mechanical ventilation	12 (10.3)	11 (4.4)	0.03
Vasopressor	16 (13.8)	15 (6)	0.01
Chemotherapy			
R-CHOP	26 (22.4)	68 (27.3)	0.31
CHOP	26 (22.4)	60 (24.1)	0.79
R-CVP	13 (11.2)	8 (3.2)	0.004
CVP	8 (6.9)	11 (4.4)	0.32
ABVD	12 (10.3)	42 (16.9)	0.11
DHAP	17 (14.7)	16 (6)	0.009
Sepsis	38 (57.6)	26 (17.2)	<0.001
Tumor lysis	18 (15.5)	10 (4.0)	<0.001
Hospital stay			
≤7 days	72 (62.1)	202 (81.1)	<0.001
>7 days	44 (37.9)	47 (18.7)	
Mortality	**17 (14.7)**	**17 (6.8)**	**0.02**

*Mann-Whitney *U* test.

**Table 3:** Factors predicting AKI.

	OR [95% CI]	*P* value
Diuretic		
No	1.0	
Yes	2.96 [1.31–6.69]	0.009
Aminoglycoside		
No	1.0	
Yes	4.75 [1.15–19.52]	0.03
Sepsis		
No	1.0	
Yes	3.76 [1.83–7.72]	<0.001
Tumor lysis syndrome		
No	1.0	
Yes	3.85 [1.54–9.59]	0.004
R-CVP		
No	1.0	
Yes	4.70 [1.20–18.36]	0.02

**Table 4:** Impact of AKI on hospital stay and mortality.

Length of hospital stay			
AKI stages 2, 3 >7 days	No AKI >7 days	Univariate analysis	Multivariate analysis
21 (48.83%)	49 (19.52%)	*P* < 0.001	OR [95% CI 2.01 (1.19, 3.40)], *P* 0.008

Mortality			
AKI	No AKI	Univariate analysis	Multivariate analysis

17 (14.7)	17 (6.8)	*P* 0.02	

**Table 5:** Factors associated with mortality in AKI.

Outcome	Death (*n* = 17)	Alive (*n* = 99)	*P* value
Age, in years	54.3 ± 16	53.6 ± 15.4	0.85
Gender			
Male (%)	8 (47.1)	74 (74.7)	0.04
Female (%)	9 (52.9)	25 (25.3)	
Tumor lysis (%)	8 (47.1)	10 (10.1)	0.001
Ventilation (%)	8 (47.1)	4 (4)	<0.001
Diuretic (%)	9 (52.9)	15 (15.2)	0.001
Vasopressor (%)	9 (52.9)	7 (7.1)	<0.001
Sepsis (%)	17 (100)	21 (42.9)	<0.001
Baseline creatinine	0.84 ± 0.22	0.84 ± 0.29	0.99
Stage of disease			
I (%)	1 (16.7)	3 (8.1)	0.50
II (%)	1 (16.7)	6 (16.2)	
III (%)	0	10 (27)	
IV (%)	4 (66.7)	18 (48.6)	

**Table 6:** Outcome of AKI at discharge.

Death	17 (14.7%)
Full recovery	45 (38.79%)
Partial recovery with no need of dialysis	40 (34.48%)
Persisting AKI with need of dialysis	14 (12.06%)

## Abbreviations

AKI: Acute kidney injury
AKIN: Acute Kidney Injury Network
R-CHOP: Rituximab-cyclophosphamide, hydroxy daunorubicin, oncovin, and prednisolone
CHOP: Cyclophosphamide, hydroxy daunorubicin, oncovin, and prednisolone
R-CVP: Rituximab, cyclophosphamide, vincristine, and prednisolone
CVP: Cyclophosphamide, vincristine, and prednisolone
ABVD: Adriamycin, bleomycin, vinblastine, and dacarbazine
DHAP: Dexamethasone, high dose Ara C, and cisplatin.
